# The complex relation between access to opioid agonist therapy and diversion of opioid medications: a case example of large-scale misuse of buprenorphine in the Czech Republic

**DOI:** 10.1186/s12954-018-0268-0

**Published:** 2018-12-04

**Authors:** Viktor Mravčík, Barbara Janíková, Barbora Drbohlavová, Petr Popov, Alessandro Pirona

**Affiliations:** 1 Department of Addictology, First Faculty of Medicine, Charles University and General University Hospital in Prague, Prague, Czech Republic; 2 National Institute of Mental Health, Klecany, Czech Republic; 3 National Monitoring Centre for Drugs and Addiction, Office of the Government of the Czech Republic, Prague, Czech Republic; 4 European Monitoring Centre for Drugs and Drug Addiction, Lisbon, Portugal

**Keywords:** Opioid substitution treatment, Buprenorphine, Diversion, Illicit market, Access, Availability, Barriers, Cost, Mortality, Crime, Opioids, Heroin

## Abstract

Opioid agonist therapy (OAT) has been available in a standard regime in the Czech Republic since 2000. Buprenorphine is the leading medication, while methadone is available only in a few specialised centres. There is an important leakage of buprenorphine onto the illicit market, and the majority of Czech opioid users are characterised by the misuse (and injecting) of diverted buprenorphine medications. Most prescribed buprenorphine for OAT is not covered by current national health insurance schemes, and patients have to pay considerable prices to afford their treatment. This affordability barrier together with limited accessibility is likely the leading factor of limited coverage of OAT and of recent stagnation in the number of patients in the official treatment programmes in the Czech Republic. It also encourages doctor shopping and the re-selling of parts of their medication at a higher price, which represents the main factor that drives the Czech illicit market for buprenorphine, but at the same time co-finances the medication of clients in official OAT programmes. Improving access to OAT by making it financially affordable is essential to further increase OAT coverage and is one of the factors that can reduce the illicit market with OAT medications.

## Introduction

Opioid dependence and its consequences represent a serious global public health concern. It is estimated that 33 million people use opioids globally, of which 16.4 million use opiates, mainly heroin, which corresponds to 0.7% and 0.4% of the world’s adult population, respectively [1]. In the European Union (EU) in 2016, the average prevalence of problem opioid use among adults was estimated at 0.4% of the population aged 15–64, the equivalent of 1.3 million long-term regular opioid users (LROUs). Heroin remains the most common opioid used among LROUs in Europe (although a steady declining trend has been observed over the last 10 years), and opioids were found in 78% of the total 7585 overdose deaths involving at least one illicit drug in the EU in 2015 [2]*.* Apart from high mortality risks, the consequences of illicit opioid use also include a high risk of infection with HIV and hepatitis through sharing of injecting equipment and engaging in risky sexual behaviours, community loss due to criminal activity, low quality of life of users and their families, economic costs and loss of social cohesion [3–5].

One of the key responses in containing and reducing the number of drug-related deaths as well as HIV infections among injecting drug users in Europe over the last 20 years has been the introduction and scaling up of opioid agonist therapy (OAT) [6]. The effectiveness of OAT has been largely evidenced for a range of outcomes including reductions in the risk of HIV and other blood-borne infections, risky sexual behaviour, risk of overdose, participation in criminal activity, illicit drug use and increased retention in treatment [7–14].

The latest European data show that an estimated 628,000 opioid users received OAT in the EU in 2016, suggesting that about 1 in 2 LROUs in the EU receive OAT. However, large variations between countries exist. In the 19 countries for which data is available, nine member states reported high OAT coverage levels above 50% of the target population; five countries report medium coverage levels (30–50%), and five countries report low coverage levels (< 30%), reaching coverage levels as low as 10% of the target population [2].

Such important differences in terms of coverage levels of OAT between EU member states [2] can largely be explained by national differences in accessibility and availability of OAT. As with any other medication, accessibility and availability of OAT impacts on how many individuals in need would have access to OAT and adhere to it. Multiple factors determine accessibility and availability of OAT, such as geographical availability, affordability for the patient; legal restrictions of who is allowed to prescribe and dispense OAT medications; and particularly in the case of OAT, rules and conditions determining who is entitled to this treatment (e.g. OAT might not be available to prisoners) and under which conditions [15–18]. In Europe, some high access OAT countries (as reflected by high coverage levels) such as Germany, Luxembourg, Ireland, Austria or France tend to share common systemic and policy features. In broad terms, drug treatment systems in these countries tend to adopt a low-threshold approach through multiple access points to OAT, with custody of drug treatment provision shared among various health care providers, including non-specialist physicians (e.g. general practitioners or family doctors) like in France or Luxembourg [2, 19]. In France for example, any general practitioner without training or specialisation can prescribe buprenorphine. To date, about 66% of the estimated 180,000 French OAT clients are prescribed their OAT exclusively by general practitioners. Additionally, policy objectives in these countries have focussed on assuring that as many opioid injectors as possible have access to OAT, retaining them in treatment and reducing the occurrence of associated harms at individual and societal level [6].

However, recent policy concerns have emerged about ease of access to OAT and lenient prescribing practices as factors contributing to the diversion of OAT medications [20]. Diversion refers to the act of redirecting opioid substitution medications from legitimate sources to illegitimate or illegal ones. Thus, an increasing number of European countries have reported a replacement of street heroin on the illicit market with synthetic opioids, including diverted opioid substitution medications. The latest European data show that in 17 European countries, about 20% of all clients entering specialised drug treatment services with an opioid problem did so for problems associated with opioids other than heroin, most commonly for problems associated with the misuse of methadone or buprenorphine [2]. The same data showed that in Finland and in the Czech Republic, more than 50% of all opioid clients entering specialised treatment sought treatment for problems due to misuse of buprenorphine.

Misuse of opioid substitution medications refers to their use outside legitimate therapeutic guidance. These medications (e.g. buprenorphine, methadone) may be misused either alone or in combination with other drugs, to attain euphoria and/or reduce cravings or withdrawal symptoms induced by opioids [21–24]. Literature reviews have shown that the burden of diversion and misuse of opioid substitution medications includes poor adherence to recommended treatment which negatively impacts treatment outcomes, excess of mortality, somatic complications associated with injecting drug use (e.g. limb ischemia, tissue necrosis) as well as increased risk of contracting blood-borne viruses such as HIV and hepatitis C, associated crime, a negative impact on prescribers’ practice or threatened reputation of treatment services and compromised public acceptance of OAT [25, 26].

Strategies have been implemented in different European countries to control and prevent the diversion of OAT medications [20]. These include providing training for clinicians and patients, implementing strategies to assure therapeutic compliance by appropriate prescription of dosing, using electronic medicine dispensers, and employing control measures such as patient toxicology tests, pill counts or unannounced monitoring. Monitoring of prescribing practices can take place through registers of patients and/or pharmacy transactions and enforcement of appropriate prescribing through disciplinary measures or administrative sanctions. Other control measures include legal restrictions on medical professions and medical points allowed to prescribe and dispense OAT medications, pre-authorisation procedures, special prescribing forms and regulations stipulating administration of doses under direct supervision in treatment centres or pharmacies.

Although increased control and regulatory oversight on the provision of OAT medications may appear intuitive to legislators, policies that excessively restrict access to treatment may in turn fuel the demand for diverted OAT medications. For example, individuals seek alternative sources to access medications for self-medication purposes and thereby increase the harms and financial burden of untreated opioid dependence [27–29]. Additionally, diversion and misuse of OAT medications are also reported in low and medium OAT coverage countries with stricter prescribing regulations and higher thresholds than the above mentioned low-threshold national OAT systems [2, 30]. Thus, the implementation of effective anti-diversion strategies while maintaining adequate access is crucial but remains particularly challenging especially that research on individual and systemic determinants of misuse and diversion of OAT medications is limited.

In order to improve our understanding of the underlying factors and determinants associated with the misuse and diversion of OAT medications at systemic level, the present overview provides a case example of a series of events and regulatory factors that have contributed to the development and maintenance of a large-scale demand for diverted OAT medications among opioid users in the Czech Republic. With its diversified, low-threshold OAT system and a significant level of OAT diversion, the Czech experience provides an important insight into the impact of large-scale diversion of opioid medications on the national drug market and its health-related consequences. It also provides valuable lessons and recommendations for policy-makers in charge of assuring access to OAT while implementing effective anti-diversion policies.

### The Czech system of opioid agonist therapy

The first unofficial OAT with ethylmorphine in the Czech Republic started in 1987, at the Addiction Treatment Centre ‘U Apolináře’ of the General University Hospital in Prague, followed by a 1-year unofficial pilot methadone programme, which was organised in 1992–1993 by the non-governmental organisation Drop-In [31]. In 1997, a governmentally approved pilot project for a methadone programme was implemented at the Addiction Treatment Centre ‘U Apolináře’ in Prague, which in 2000 was rolled out to the rest of the country in a standard regime [32]. In 2006, OAT was introduced in two pilot prisons and became available in custodial settings in standard regime from 2008 onwards with 10 out of 35 prisons having permission to provide OAT. However, OAT cannot be initiated in Czech prisons but only continued if the inmate was already on OAT at entry.

The procedures for OAT are defined in the Standard for Substitution Treatment [33]. Substitution preparations are only administered orally. There are three OAT preparations currently available in the Czech Republic: (1) methadone (since 1997), prepared from an imported generic substance as a magistral preparation; (2) mono-buprenorphine preparations Subutex® since 2000, and later Addnok®, Buprenorphine Alkaloid®, and Ravata®; (3) a composite preparation Suboxone® containing buprenorphine and naloxone (since 2008). The function of the naloxone component in Suboxone® is to deter intravenous abuse as parenteral administration rapidly induces opioid withdrawal symptoms, while regular, intended use does not (as naloxone is minimally bioavailable with sublingual ingestion) [34], although real-life data show that there is injecting of Suboxone® ongoing among opioid users and that its safety profile is not necessarily superior of the buprenorphine alone [35, 36].

OAT medications can be prescribed by any physician regardless of the specialisation, but under a stricter prescription regime applied for prescribing opioid analgesics and medicinal products containing narcotic substances. There are 12 specialised treatment centres in the Czech Republic that provide primarily methadone treatment (but do also provide buprenorphine) and 51 officially registered non-specialised units such as general psychiatry practices or general practitioner practices prescribe exclusively buprenorphine. In reality, there are an additional unknown number of non-specialised physician practices that also prescribe buprenorphine in the country.

All physicians administering an opioid substitution medication are obliged by law to report each individual patient to the substitution treatment register (STR) which is managed by the Institute of Health Information and Statistics. This register operates since 2000 and until 2007; it was collecting patient data only from specialised Substitution Treatment Centres administering methadone and only through paper-based forms. From 2007 onwards, forms are submitted electronically via a web-based application which collects data from all specialised as well as non-specialised centres including those prescribing buprenorphine. Since 2015, STR has been integrated into a newly established National addiction treatment register.

**Fig. 1:**
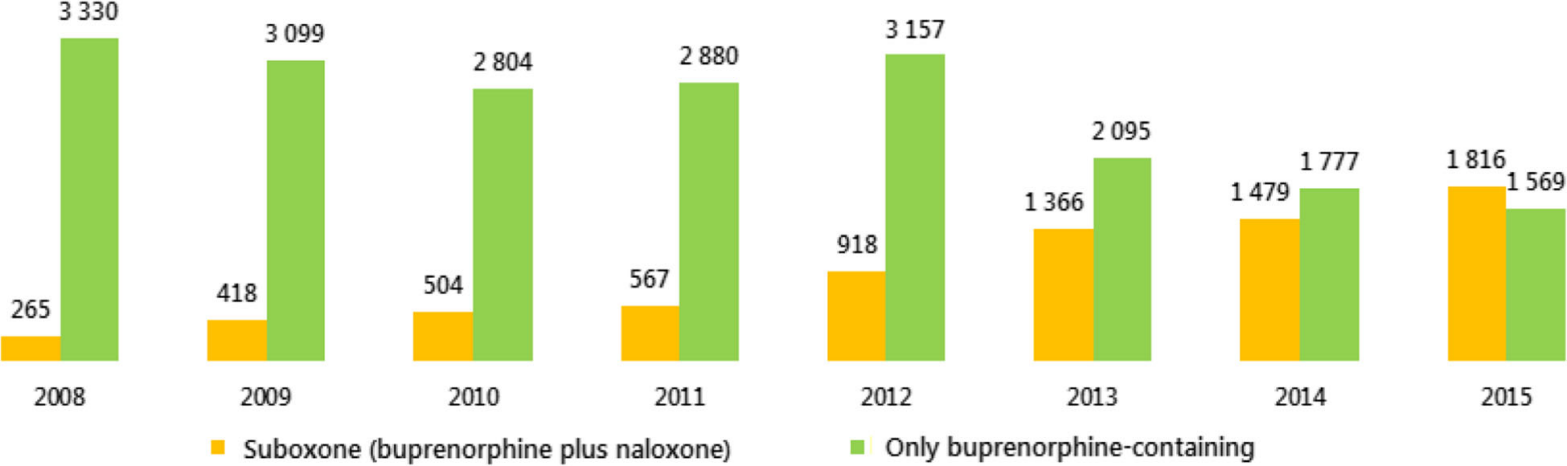
Amount of buprenorphine distributed in buprenorphine-only and buprenorphine plus naloxone medications, 2008–2015 (in grams). Source: Ministry of Health in Mravčík et al. [37]

Proprietary medicinal formulation containing methadone has not yet been marketed in the Czech Republic. Between 10 and 20 kg of pure methadone were annually imported, and 3 to 4 kg of buprenorphine in OAT preparations were distributed annually in the Czech Republic in the last 10 years (Table 1). Since the introduction of Suboxone® in 2008, the share of buprenorphine used in the composite medication Suboxone® has increased steadily with a corresponding decline in preparations containing uniquely buprenorphine (Fig. 1), which has probably twofold reasons: the lower price of Suboxone® for patients (see below more details) and its increased prescriptions due to better pharmacological profile reducing risk of injecting use. In 2015, 54% of the amount of buprenorphine was distributed in Suboxone® and 46% in mono-preparations [37].

**Table 1:** Amounts of substitution drugs imported (methadone) and distributed (buprenorphine), 1999–2015

Year	Methadone—import (kg)	Buprenorphine—distribution (kg)
1999	13.5	–
2000	11.7	0.0235
2001	0.0	0.0862
2002	0.0	0.5098
2003	8.1	1.3094
2004	11.3	2.2219
2005	5.7	2.9573
2006	12.2	3.4143
2007	10.8	3.3150
2008	12.6	3.5945
2009	15.4	3.5170
2010	22.5	3.3080
2011	24.3	3.4468
2012	18.0	4.0751
2013	17.9	3.4607
2014	16.3	3.2563
2015	16.4	3.3848

Source: Ministry of Health in Mravčík et al. [37]

**Fig. 2:**
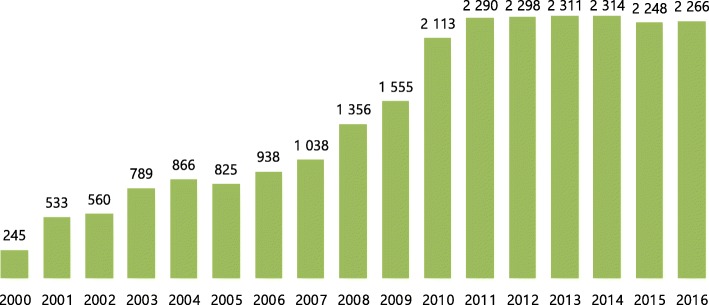
Number of clients registered in OAT, 2002–2016. Source: Institute of Health Information and Statistics of the Czech Republic in Mravčík et al. [37]

In 2016, 63 centres reported 2266 patients to the STR. There was an increase in number of patients by 300% between 2002 and 2016; however, there is a stagnation since 2011 (see Fig. 2).

Of 2266 reported patients, 688 (30%) were prescribed methadone and 1578 (70%) were prescribed buprenorphine preparations (of them 44% Suboxone® and 56% buprenorphine mono-preparations). About 95% of these patients are reported from outpatient psychiatric facilities including specialised Substitution Treatment Centres [37, 38]. Despite the law, not all physicians who prescribe OAT report to the SRT and not all patients are registered. Based on the survey among general practitioners, it is estimated that an additional 1600 patients are prescribed buprenorphine preparations [38]. Thus, the total number of individuals in OAT in the Czech Republic can be estimated at about 4000 individuals annually, of them 700 on methadone (since all methadone centres report to the register), and (based on the proportion of reported) more than 1400 on Suboxone® and nearly 2000 on mono-buprenorphine preparations.

### Misuse of OAT medications and associated harms in the Czech Republic

There is a considerable leakage of buprenorphine preparations onto the illicit market in the Czech Republic, while diversion of methadone is almost non-existent since methadone is not available in pharmacies as a proprietary medicinal product [39, 40]. To date, the majority of estimated amount of LROUs (approximately 60%) in the Czech Republic are individuals misusing buprenorphine, using it outside a therapeutic context, mostly injecting it [40].

**Fig. 3:**
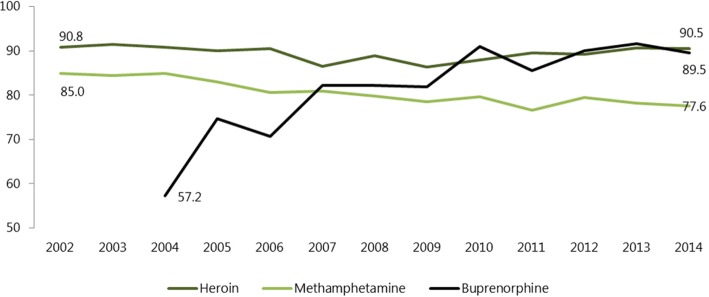
Proportion of people who inject drugs (PWID) by primary drug reported to the Treatment Demand Register, 2002–2014 (in %). Source: Füleová et al. [41]

Methamphetamine remains however in the centre of drug problem in the Czech Republic with an estimated 34,300 long-term regular methamphetamine users in 2016. The number of LROUs was estimated at 12,500 individuals (95% CI 12000–12,900) of whom 3400 (95% CI 3200–3600) use heroin as primary drug and 7300 (95% CI 7000–7600) use buprenorphine as primary drug [40] (Table 2). Both opioids are mostly injected (Fig. 3) [41]. Polydrug use is common among long-term regular drug users in the Czech Republic with the most prevalent combination of methamphetamine and buprenorphine as the primary drugs [42, 43]. Finally, opioid use in the Czech Republic is also characterised, by an increasing misuse of opioid analgesics diverted from medical sources such as fentanyl, codeine, or morphine and, to a much lesser extent, by the use of raw opium from poppy fields [40, 44, 45]. Trends data on the estimated number of LROUs by primary drug clearly indicates a progressive shift from heroin use to misuse of buprenorphine between 2006 and 2016 (Table 2).

**Table 2:** Estimated number of long-term regular opioid users (central values) in the Czech Republic between 2006 and 2016

Year	Heroin	Buprenorphine	Other opioids	Total	Total per 1000 population (aged 15–64)
2006	6200	4300	–	10,500	1.44
2007	5750	4250	–	10,000	1.36
2008	6400	4900	–	11,300	1.52
2009	7100	5100	–	12,100	1.63
2010	6000	5000	–	11,000	1.48
2011	4700	4600	–	9300	1.27
2012	4300	6300	–	10,600	1.47
2013	3500	7200	–	10,700	1.50
2014	4100	7200	–	11,300	1.59
2015	4300	7300	1100	12,700	1.81
2016	3400	7300	1700	12,500	1.79

Source: Mravčík et al. [40]

Buprenorphine injection is more frequent than heroin injection since there is a phenomenon of division of buprenorphine tablets into smaller pieces (such as one quarter of an 8 mg tablet or even less) injected little by little and Czech buprenorphine users consume nearly 30 syringes (injecting sets) a week on average [46].

### Public health impacts of use of OAT medications

Despite the high-risk prevalence of injecting drug use, HIV prevalence among PWIDs is below 1% and HCV prevalence in this population in the Czech Republic is among the lowest in the EU [30] varying from 20% in low-threshold settings to around 50% in OAT or prisons [37]. The containment of blood-borne diseases among IDUs is probably associated with high coverage of harm reduction programmes including needle and syringe programmes, especially in Prague where the large majority of LROUs are located [37].

**Fig. 4:**
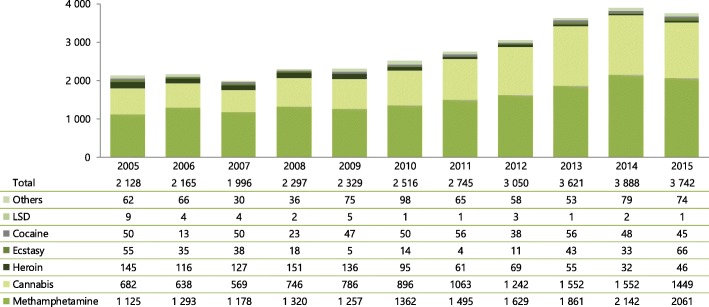
Number of persons investigated for drug-related crime offences by primary drug, 2005–2015. Source: National Drug Squad of the Police of the Czech Republic in Mravčík et al. [37]

Since the early 2000s, positive public health impacts were observed with increased provision of OAT, in parallel with an increase in diverted buprenorphine in the open drug scene, that are still apparent today [47]. Thus, a considerable decrease in opioid-related fatal overdoses has been observed since 2001 from over 50 cases to 10–20 cases annually (Table 3) while a recent increase is likely attributed to an increased misuse of opioid analgesics. Buprenorphine was mentioned in toxicological reports of two cases ever: in 2011 in combination with methadone, THC and alprazolam, and in 2012 in combination with morphine (likely metabolite of heroin), methamphetamine and THC [48, 49]. Overall, the Czech Republic with 6 deaths per million has one of the lowest direct drug-induced death rates in the EU [2]. At the same time, there has also been a substantial reduction of street heroin and a decrease of heroin-related drug crime offences in an overall context of increase in the total number of drug-related crime offences between 2002 and 2015, primarily due to cannabis- and methamphetamine-related offences (Fig. 4).

**Table 3:** Fatal overdoses by illicit drugs and volatile substances in the Czech Republic between 2001 and 2015

Drug	2001	2002	2003	2004	2005	2006	2007	2008	2009	2010	2011	2012	2015*
Opiates/opioids	56	21	21	19	24	10	14	15	20	19	6	12	20
Volatile substances	15	14	22	20	18	14	14	10	8	16	4	10	7
Methamphetamine	5	8	9	16	14	12	11	19	18	18	16	16	15
Cocaine	0	0	0	1	1	1	1	0	0	0	1	0	1
Other synthetic drugs	0	0	1	0	2	0	0	0	3	2	1	0	0
Unspecified	8	1	2	1	3	5	0	0	0	0	0	0	1
Total illicit drugs and inhalants	84	44	55	57	62	42	40	44	49	55	28	38	44

Source: Institute for Health Information and Statistics, National Monitoring Centre for Drugs and Addiction and The Czech Society for Legal Medicine and Forensic Toxicology in Mravčík et al. [37]. Note: In 2015, the Special drug mortality register based on reporting from forensic medicine departments was transformed into newly established National Register of forensic autopsies and toxicological examinations administered by Institute for Health Information and Statistics. Data from 2013 and 2014 are not available

## Factors driving the illicit market with buprenorphine medications

### Legislative and administrative factors

The first reports of buprenorphine (Subutex®) on the illicit market in Prague emerged in the summer of 2002 from outreach programmes [50], while at the end of 2002, similar reports began to emerge in northern Bohemia and sporadically elsewhere in the country. One of the factors that contributed to making the diversion of buprenorphine more widespread was the amendment to Act No. 167/1998 Coll., on addictive substances, which came into effect on 1 September 2003, rescheduling the classification of buprenorphine into a stricter prescription regime requiring, among other changes, the use of special prescribing forms for controlled substances. This transition led to a temporary gap in the availability of this medication in medical practices and created an unexpected demand within the illicit market for buprenorphine-based products [39]. The use of special prescribing forms is known to potentially impede the accessibility of controlled medicines if these are not readily available, entail a range of administrative requirements for healthcare professionals and/or are not free of charge [51]. During the same period, there was also a shortage in the supply of Subutex® to the Czech Republic which further decreased availability of this medication and contributed to the increasing demand on the illicit market [39]. As the illicit market of buprenorphine grew, a spillage effect was observed with injecting opioid users gradually moving onto injecting buprenorphine instead of heroin, which was and still is significantly more expensive [42, 45, 52].

Registration of physicians and patients participating in OAT in STR was introduced from the beginning to prevent double prescription and doctor shopping [39]. However, this legal obligation has not been fully endorsed and noncompliance is not sanctioned in practice by state authorities. Moreover, since the majority of buprenorphine purchases in pharmacies are paid directly by patients, prescription practices are not subject of any control from health insurance agencies as it would be the case if buprenorphine was covered by health insurance schemes (see also below the section on affordability).

### Geographical availability

Availability of OAT in the Czech Republic remains low with some geographical differences. In total, the coverage of OAT is below 20% according to STR. Especially in Prague, where more than 70% of the total estimated number of LROUs in the country are located, the availability and coverage of OAT is limited—only 14.8% of the estimated number of LROUs in Prague are registered in OAT with one OAT provider for every 445 estimated LROUs in the capital city. Despite lower numbers of LROUs in other regions of the country, the coverage of OAT in 8 out of 13 regions remains sub-optimal (< 30% of the target population) (Table 4).

**Table 4:** Estimated number of long-term regular opioid users (LROUs), registered OAT clients, number of OAT providers and LROUs/OAT providers (availability) and number of OAT patients as a percentage of the estimated number of LROUs (coverage) by region and total for the Czech Republic in 2016

Region	Number of estimated LROUs	Number of registered OAT clients	Number of registered OAT providers	Number of estimated LROUs per 1 provider	Proportion of estimated LROUs in OAT
Prague	8900	1317	20	445	14.8
Central Bohemia	700	138	8	88	19.7
South Bohemia	500	52	5	100	10.4
Pilsen	400	41	3	133	10.3
Karlovy Vary	100	7	1	100	7.0
Ústí nad Labem	900	299	5	180	33.2
Liberec	< 50	22	3	17	44.0
Hradec Králové	200	105	3	67	52.5
Pardubice	< 50	0	0	0	0.0
Vysočina	< 50	20	2	25	40.0
South Moravia	400	142	7	57	35.5
Olomouc	100	42	1	100	42.0
Zlín	100	1	1	100	1.0
Moravia-Silesia	300	80	4	75	26.7
Czech Republic in total	12,500	2266	63	198	18.1

Source: Mravčík et al. [40]

### Affordability

One important factor contributing to the high-level diversion of buprenorphine in the Czech Republic is the cost and affordability of buprenorphine-based OAT medications for patients. Although not covered by Czech health insurance schemes, methadone is free of charge for patients since the import of the generic substance is financed directly by the Ministry of Health. However, geographical availability of methadone is low since it is available only in 12 specialised OAT centres throughout the country (and in prisons). Mono-buprenorphine preparations are also not covered by the national health insurance schemes, and patients have to pay the total price when purchased in pharmacies, which for example for Subutex® corresponds to CZK 1450 (55€/$62) for a package of seven tablets of 8 mg. On the other hand, Suboxone®, which costs CZK 460 (17€/$20) for a package of seven tablets of 8 mg in 2017, is fully covered by the health insurance since 2010. However, the process and criteria for the reimbursement of this medication are cumbersome. Suboxone® is only reimbursed if it is prescribed exclusively by a psychiatrist or an addiction medicine specialist and a special bilateral contract is required between the insurance company and the prescriber. Furthermore, the medication for the patient is purchased and dispensed by the treatment centre (not by the community pharmacy) and the costs are then reimbursed to the treatment centre. Importantly, the insurance company does not reimburse the medication in case of non-adherence of the patient.

As a result, just a very small proportion of clients are treated with reimbursed (and thereby free of charge) medications. According to the biggest insurance company (VZP), Suboxone® was financed for 157 clients in the whole country in 2014. According to the survey carried out in OAT centres in 2015, Suboxone® was financed for approximately 75 clients in 4 centres [53]. Both figures mean that the majority of clients on buprenorphine (more than 3000 individuals) have to pay the full price of their medication—calculated for 8 mg/day, corresponding to 70€/$80 for Suboxone® and 220€/$250 for mono-preparations per month; in flexible dosing schedule reaching daily 12 mg and more, the costs can exceed 105€/$120 for Suboxone and 330€/$370 for mono-preparations per month. Adequate dosing is an important issue for treatment effectiveness. Higher doses improve the treatment retention and reduce illicit opioid use [54, 55], and titrated doses of buprenorphine from 12 up to 32 mg daily may offer better treatment outcome for patients who would not respond to the low or moderate doses [56–58].

Considering the average monthly wage in the Czech Republic of CZK 28,000 in 2016 [59] (1050€/$1200) and the legal minimum monthly wage of CZK 9900 in 2016 [60] (370€/$420), it becomes apparent that the affordability of this treatment, especially at effective therapeutic dosages, constitutes a major barrier in terms of accessibility for LROUs. As an immediate result of this barrier, doctor shopping takes places with the aim to obtain prescriptions of higher amounts in order to re-sell some of the medications on the illicit market at a higher price in order to finance one’s own medication [39, 44]. This practice in the Czech Republic was already observed and described in 2004 [52]. The price of a single 8 mg tablet of Subutex® bought at a pharmacy is approx. CZK 200 (7.5€/$8.2); on the illicit market, the price is usually double that price, but when buprenorphine is temporarily unavailable (like for example in Prague in April and May 2012), the price can be up to six times higher [38, 48]. Similar high prices on the illicit market are observed for Suboxone®.

A possible consequence of the high price of licit and illicit buprenorphine is an overall sub-optimal dosing. The reported daily dose of buprenorphine among users of buprenorphine who are clients of outreach programmes in Prague reaches usually between 2 and 4 mg [44, 61]. The consequences of sub-optimal dosing are well known and are associated with polyuse of licit and illicit drugs (e.g. [62]).

Finally, the overall retail price of annually consumed amounts of buprenorphine in the Czech Republic with current dosing (providing 50:50 rate between Suboxone® and mono-buprenorphine preparations) reaches at least CZK 60 million (2.2 million €/$2.4 million)—the totality being nearly all paid and covered by the patients themselves.

## Discussion

The present overview of the diversion and misuse of OAT in the Czech Republic indicates that limited geographical availability of OAT coupled with low affordability of buprenorphine-based medications constitute significant barriers for LROUs to access high quality and professionally delivered OAT. It is important to acknowledge that this overview is a mere descriptive analysis of the national situation based on existing epidemiological data and published studies with no possibility to establish any causal relationship between the different factors explored in this overview. A number of other contributing factors, such as social, cultural or drug market-related factors not explored here, may certainly have also contributed to this phenomenon. The associations between events put forward here and their potential consequences on public health could however provide directions for future research in this field to validate these findings.

Scientific literature shows that restrictive reimbursement conditions for OAT within national health insurance schemes and complicated administrative and regulatory frameworks for OAT provision are considered important factors in low access to and low coverage of OAT [51, 63–65]. Similarly, limited access to buprenorphine-based medications in the Czech Republic is associated with cumbersome and selective reimbursement schemes which result in less than 5% of the target population receiving this specific medication free-of charge. The out of pocket cost for medicine within flexible dosing on the other hand can exceed the rate of the minimum legal wage in the Czech Republic. Considering the disadvantaged socio-demographic profile of LROUs and PWIDs, this significant economic barrier may encourage doctor shopping and the re-selling of parts of their medication at inflated prices for self-medication purposes (e.g. reducing craving and withdrawal symptoms), thereby driving the diversion and supply of buprenorphine outside the therapeutic context onto the Czech illicit drug market. Self-medication due to restricted access to treatment is a frequent motivator for diversion and misuse, and this is consistent with published data from other countries [66].

Furthermore, current literature on responses to misuse and diversion of OAT focus primarily on improving treatment quality (e.g. optimal dosing), supervision of patients (urine testing, supervised dispensing), monitoring of prescribing practices to avoid doctor shopping and greater availability of abuse-deterrent formulations such as the buprenorphine-naloxone composite [20, 26]. These anti-diversion measures however assume that a majority of LROUs are effectively accessing OAT and that abuse-deterrent medications, such as Suboxone®, are not largely available. Data presented here show that in most Czech regions, including in the capital city where most LROUs are located, coverage levels of OAT barely reach 10–30% of the target population (even when accounting for unregistered OAT clients). Stricter anti-diversion policies may seem an intuitive choice when facing large-scale diversion and misuse of OAT medications as experienced in the Czech Republic. However, it is evident that further limiting access to OAT is unlikely to have a positive impact on the current situation. A policy that aims at increasing availability and facilitating access to buprenorphine-based medications by removing structural barriers linked to legal prescribing requirements, increasing affordability and geographical coverage of OAT may represent an alternative solution [67]. Thus, increasing access to OAT as an anti-diversion strategy may appear counter-intuitive, but could be considered, based on an informed and well-researched analysis of the determinants, in countries with limited access to OAT and significant diversion problems. Evolution of the levels of diversion and associated harms, as well as intake into established OAT should subsequently be closely monitored.

There is an increasing number of European countries reporting drug-related deaths involving diverted opioid substitution medications such as methadone and buprenorphine [2]. Greater scrutiny on prescribing practices and improved treatment adherence may reverse this negative trend associated with misuse of OAT reported in these countries, as well as the range of negative health consequences ranging from poor health, transmission of blood-borne viruses to increased mortality rates. A decreasing rate of opioid-induced mortality was observed in the Czech Republic during the same period when a significant shift from heroin to diverted buprenorphine on the illicit market occurred. Research is needed to explore the relation between these two parallel trends and how reduced use of heroin, with an increasing illicit use of buprenorphine among the Czech high-risk opioid using population may have contributed to a decrease in opioid-related mortality. On the other hand, the opposite is however observed in Finland where the decrease in heroin supply resulted in a significant increase in diverted buprenorphine supply between 2000 and 2004, which resulted in a sharp decrease in heroin-related deaths and a sharp increase in death rates associated with misuse of buprenorphine during the same period [68]. Similar phenomena with opposite consequences in the Czech Republic and in Finland require further investigation on the main determinants explaining these differences.

Finally, our observations are in line with the conventions and treaties enforcing human rights and right to health in the field of opioid dependence treatment. The latest international guiding principles for legislation and regulation of opioid agonist treatment issued by Pompidou Group [67] emphasise that from a normative point of view, anyone with a diagnosis of dependence syndrome must have access to treatment based on the latest scientific and medical knowledge, that obligations for physicians should be limited to what is strictly necessary and proportionate to ensure the effectiveness of the treatment and its security to third parties and that authorities should ensure that treatment is paid for and that healthcare professionals are duly remunerated.

In conclusion, the negative consequences associated with the illicit opioid market in the Czech Republic could be further reduced and further public health benefits at population level could be achieved through improved access, affordability and quality of OAT provision for the majority of opioid-dependent individuals.

## Abbreviations

CZK: Czech crown
EU: European Union
LROUs: Long-term regular opioid users
OAT: Opioid agonist therapy
STR: Substitution treatment register
